# The Anticancer Potential of Edible Mushrooms: A Review of Selected Species from Roztocze, Poland

**DOI:** 10.3390/nu16172849

**Published:** 2024-08-26

**Authors:** Piotr Roszczenko, Olga Klaudia Szewczyk-Roszczenko, Agnieszka Gornowicz, Iga Anna Iwańska, Krzysztof Bielawski, Monika Wujec, Anna Bielawska

**Affiliations:** 1 Department of Biotechnology, Medical University of Bialystok, Kilinskiego 1, 15-089 Bialystok, Poland; piotr.roszczenko@sd.umb.edu.pl (P.R.); agnieszka.gornowicz@umb.edu.pl (A.G.); igaannaiwanska@gmail.com (I.A.I.); 2 Department of Synthesis and Technology of Drugs, Medical University of Bialystok, Kilinskiego 1, 15-089 Bialystok, Poland; olga.szewczyk@sd.umb.edu.pl (O.K.S.-R.); krzysztof.bielawski@umb.edu.pl (K.B.); 3 Department of Organic Chemistry, Faculty of Pharmacy, Medical University of Lublin, 4A Chodzki Street, 20-093 Lublin, Poland

**Keywords:** edible mushrooms, Poland, anticancer treatment, bioactive compounds, mushroom extracts, fungal metabolites, cancer therapy, medicinal fungi

## Abstract

Edible mushrooms are not only a valued culinary ingredient but also have several potential medicinal and industrial applications. They are a rich source of protein, fiber, vitamins, minerals, and bioactive compounds such as polysaccharides and terpenoids, and thus have the capacity to support human health. Some species have been shown to have antioxidant, anti-inflammatory, anticancer, and immunomodulatory properties. We have therefore attempted to summarize the potential properties of the edible mushrooms popular in Poland, in the Roztocze area.

## 1. Introduction

Natural sources are promising for development of treatment options for different diseases. In the pharmaceutical industry, there are plenty of medications that are present in nature, e.g., doxorubicin, cisplatin, and digoxin. Mushrooms have long been appreciated for their unique flavors and textures, but in recent years, their nutritional and medicinal benefits have also come into the spotlight [1].

Nutritionally, mushrooms are quite remarkable. They are low in calories and fat, making them an excellent choice for those trying to manage their weight. Despite their low calorie content, mushrooms are packed with essential nutrients. They are a good source of vitamins, particularly B vitamins such as riboflavin, niacin, and pantothenic acid, which play vital roles in energy production and overall cell function. Mushrooms also provide important minerals such as selenium, copper, and potassium, which contribute to various bodily functions, including immune support, antioxidant protection, and maintaining proper heart and muscle function [2].

One of the outstanding features of mushrooms is their fiber content, specifically beta-glucans. Beta-glucans are known for their immunomodulatory properties, helping to boost the immune system. In addition, these fibers can aid digestion and improve gut health by acting as prebiotics that support the growth of beneficial gut bacteria [3,4].

Mushrooms also contain a unique antioxidant called ergothioneine, which is not found in many foods. Ergothioneine has been linked to protecting cells from damage and reducing inflammation, which may help prevent chronic disease [5,6]. Another notable compound found in certain mushrooms, such as shiitake and maitake, is lentinan, which has shown potential in supporting the immune system and fighting cancer cells [7].

In addition to their nutritional value, mushrooms have a long history of use in traditional medicine. Many species have been studied for their therapeutic properties. Reishi mushrooms (*Ganoderma lucidum*), for example, are renowned for their ability to boost the immune system, reduce stress, and improve sleep. They are often used in herbal medicine to support general health and longevity [8,9]. Turkey tail mushrooms (*Trametes versicolor*) contain polysaccharide K (PSK), which is used in some countries as an adjuvant therapy in cancer treatment due to its immune-boosting properties [10,11,12].

Lion’s mane mushrooms (*Hericium erinaceus*) have gained attention for their potential to support brain health. They contain compounds that can stimulate brain cell growth and improve cognitive function, making them of interest in research into neurodegenerative diseases such as Alzheimer’s disease [13].

The perception of mushrooms as a nutritious and medicinal food has evolved because of these findings. It is especially important in the context of the cancer epidemic, which is seen today. In 2022 alone, cancer was the immediate cause of 9,743,832 deaths estimated by the WHO [14]. There is still a need for new, safer therapies due to the rising resistance to chemotherapy and the burden-bearing side effects of commercially used medications [15]. This is the reason for the search for new therapies among other natural sources like mushrooms. In this review, we summarized findings about compounds and extracts with anticancer potential.

## 2. Mushrooms from Polish Forests as a Potential Source of Anticancer Compounds: Current Research and Findings

**Figure 1:**
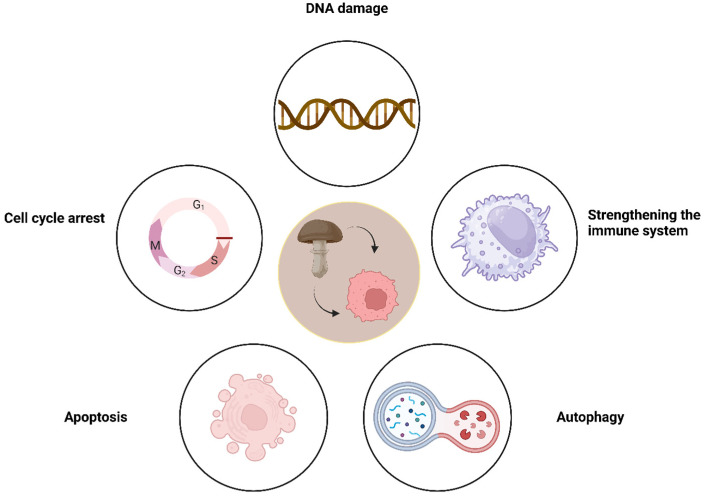
Mechanisms of the anticancer actions of mushroom-derived products.

This chapter discusses selected species of edible mushrooms found in Roztocze, a remarkable geographic region with exceptional natural, cultural, and historical value. It is characterized by dense forests, silence, clean air, and a lack of industry. Well-preserved areas are under protection and many species of plants and endangered animals have their habitats there. The most studied and utilized products are extracts from the fruiting bodies of these mushrooms. However, research into their anticancer effects frequently involves polysaccharides, proteins, and even mRNAs. Scientific reports on mushroom extracts and bioactive mushroom compounds with potential applications in anticancer therapy are reviewed. Research has demonstrated that these products can activate various mechanisms, including apoptosis, autophagy, cell cycle arrest, DNA damage, and enhanced immune system activity (Figure 1).

### 2.1. Order Boletales

#### 2.1.1. *Boletus edulis*

**Figure 2:**
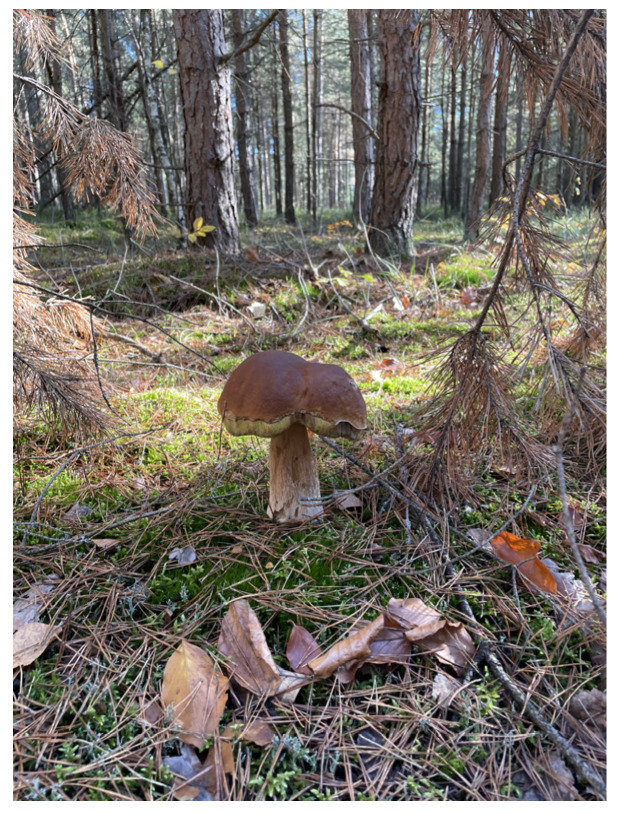
*Boletus edulis*.

The “Noble Boletus” (Figure 2), a very popular edible mushroom found wild in forests all over the world, is known not only for its typical appearance—a brown, bun-shaped cap and a white reticulated stem—but also for its flavor and wide pharmacological activities. It has been recognized for its antidiabetic, antioxidant, anti-inflammatory, antimicrobial, and anticancer activities [16]. Reports on the anticancer activity of *Boletus edulis* species will be summarized in this chapter.

##### Polysaccharides

Lemieszek et al. used hot-water extraction and ion exchange chromatography to obtain five biopolymer fractions from the fungus *Boletus edulis*. These fractions were not toxic to human colon epithelial cells, as shown by the LDH assay. Conversely, the cytotoxicity test on the human colon carcinoma LS180 cell line, conducted using the MTT assay, revealed that three fractions (BE3, BE4, and BE5) had significant activity, with IC_50_ values of 1.00 mg/mL, 15.80 mg/mL, and 45.4 mg/mL, respectively. This activity was further confirmed using the BrdU assay, which showed IC_50_ values for the reduction in the proliferation of the LS180 cell line to be 10.3 μg/mL, 308 μg/mL, and 595.5 μg/mL, respectively [17]. The study was extended to the HT-29 colorectal adenocarcinoma cell line, where the IC_50_ values from the MTT assay after 96 h of treatment were found to be 26.8 µg/mL, 47.4 µg/mL, and 45.4 µg/mL for the respective biopolymer fractions. [18]. Due to its potent activity, the BE3 fraction was selected for further studies. BE3 was shown to induce cell accumulation in G0/G1 phase. Additionally, it was found to modulate the p16/cyclin D1/CDK4-6/pRb pathway [17]. In a further series of experiments, the researchers discovered that the fraction obtained consisted mainly of ribonucleic acid, and they investigated its pro-apoptotic activity. They observed that the fraction induced apoptotic death of cancer cell lines. BE3 enhanced caspase activity and caused an increase in cytosolic nucleosomal fragments. The mechanism of apoptosis was confirmed by increased expression levels of the pro-apoptotic proteins Bax, TP53 and CDKN1A, and a decrease in the anti-apoptotic protein Bcl-2 [19]. The researchers also confirmed that the antiproliferative ability of the BE3 fraction in HT-29 cells depended on the modulation of cell cycle regulatory proteins (cyclin D1, cyclin A, p21, and p27). Additionally, the action of the BE3 fraction in combination with MAPK/Erk pathway inhibitors demonstrated additive activity; the fraction itself influenced the silencing of MAPK/Erk pathway signal transduction, as evidenced by a decrease in the phosphorylation levels of components of the cascade [18].

Meng et al. isolated a cold-water-soluble polysaccharide (BEP) from the fruiting body of *Boletus edulis*, which is mainly composed of mannose, glucuronic acid, and galacturonic acid. They confirmed its anticancer activity on the human breast cancer cell line MDA-MB-231 and mouse breast cancer cell line Ca761, with IC_50_ values for the MTT assay of 152.76 μg/mL and 134.55 μg/mL after 24 h treatment, and 120.62 μg/mL and 105.37 μg/mL after 48 h, respectively. Furthermore, they demonstrated that BEP induces apoptosis through the mitochondrial apoptosis pathway in a concentration-dependent manner. This was evidenced by an increase in cytochrome C levels in the cytosol of cells, an increase in the level of the pro-apoptotic protein Bax, and a decrease in the level of the anti-apoptotic protein Bcl-2. This process appears to be triggered by an intracellular increase in ROS-mediated damage. Additionally, cell cycle inhibition was observed, with the polysaccharide causing MDA-MB-231 cells to be blocked in S phase and Ca761 cells to be blocked in G0/G1 phase [20].

A water-soluble polysaccharide isolated from the fruiting bodies of *Boletus edulis* (BEP), averaging 113.432 Da and composed of 93.4% carbohydrates, including glucose, galactose, rhamnose, and arabinose, demonstrated antitumor activity in mouse studies in vivo. In these studies, BEP at a dose of 400 mg/kg reduced the growth of transplanted mouse renal cancer cells (Renca) over 32 days of oral administration, showing efficacy comparable to injectable 5-fluorouracil at 20 mg/kg. Additionally, BEP exhibited no cytotoxic effects on the hematopoietic system, liver, or kidneys, unlike 5-fluorouracil. BEP also enhanced IL-2 and TNF-α secretion, stimulated splenocyte proliferation, and increased the activities of natural killer (NK) cells and cytotoxic T lymphocytes (CTL), which may contribute to its antitumor effects [21].

##### Proteins

On the other hand, Zhang et al. isolated a new anti-cancer protein from the dried fruiting bodies of the Noble Boletus, named BEAP. This protein induces apoptosis in A549 non-small-cell lung cancer cells after 48 h of incubation. It was observed that treatment with BEAP led to decreases in PARP and caspase-8 levels, increases in the expression of the pro-apoptotic proteins Bid and Bax, and a decrease in the expression of the anti-apoptotic protein Bcl-2. BEAP also inhibited cell migration, blocked the cell cycle in G1 phase, and inhibited malignant proliferation with an IC_50_ value of 75 μg/mL. Notably, BEAP showed no cytotoxicity to the human embryonic kidney cell line HEK293 [22]. Further studies of the protein confirmed that BEAP not only induces apoptosis but also triggers paraptosis and autophagy. In A549 cells, after 12 h of incubation, the process of paraptosis was observed, as evidenced by decreases in the expression of the negative regulators of paraptosis Alix and caspase-9, along with the activation of the MAPK pathway and increased expression of the *p*-ERK, *p*-JNK and P_38_ proteins. Additionally, the process of autophagy was confirmed within 48 h by decreases in the expression of proteins involved in autophagy (AMPK and mTOR) and growth (BECN1 and DRAM) [23].

Bovi et al. isolated lectins with antiproliferative activity from the fruiting body of *Boletus edulis.* The homotetrameric lectin reduced the incorporation of [^3^H]-thymidine into DNA in various cell lines, with the strongest effect observed on the colon cancer cell line HT29, showing 92% inhibition at a concentration of 10 µg/mL. The lectin also inhibited breast adenocarcinoma MCF-7 cells by 77% and the liver cancer cell line HepG2 by 79% at the same concentration [24]. In a subsequent study, the researchers isolated and purified homodimeric lectin folded as β-trefoil domain structure. This lectin demonstrated cytotoxic activity against four cell lines in the MTT assay after 24 h of incubation: MCF-7, HepG-2, colorectal adenocarcinoma CaCo-2 and pancreatic duct adenocarcinoma CFPAC-1 cells. Additionally, in the [^3^H]-thymidine incorporation assay, the lectin showed notable activity against cervical adenocarcinoma HeLa, glioblastoma U-87 MG, HT-29, melanoma SK-MEL-28 and lung adenocarcinoma A549 cells. The best antitumor results were observed in the HepG2 line (IC_50_ of approximately 3 µg/mL for the MTT assay) and HeLa and U-87 MG cells (IC_50_ values of about 4 µg/mL and 2.5 µg/mL, respectively, for the [^3^H]-thymidine incorporation assay) [25].

##### Nanoparticles

Kaplan et al. synthesized gold nanoparticles (BE-AgNPs) from an aqueous solution of the fungus *Boletus edulis* using green chemistry. The product demonstrated significant cytotoxic effects on several cancer cell lines, including breast adenocarcinoma MCF-7, colorectal adenocarcinoma HT-29, and hepatocellular carcinoma HUH-7 cells, with IC_50_ values of 20.25 μg/mL, 21.55 μg/mL, and 6.55 μg/mL, respectively, after 24 h of incubation, and 8.48 μg/mL, 8.00 μg/mL, and 3.59 μg/mL after 48 h incubation. Furthermore, the nanoparticles exhibited a wound healing ability at low concentrations (1.25 μg/mL and 2.5 μg/mL) on murine fibroblast cells L929 [26].

##### Extracts

Kosanić et al. investigated a methanol extract from *Boletus edulis*, finding it to exhibit relatively high antitumor activity with IC_50_ values of 35.7 μg/mL, 34.91 μg/mL, and 48.09 μg/mL for cervical adenocarcinoma HeLa cells, human lung carcinoma A549 cells and human colon carcinoma LS174 cells, respectively, in the MTT assay after 72 h of incubation. The extract did not exceed toxic levels of metals. It also demonstrated antioxidant and antimicrobial properties, inhibiting various bacterial and fungal species [27].

#### 2.1.2. *Boletus reticulatus Schaeff*

*Boletus reticulatus* Schaeff, a fungus in the *Boletaceae* family, is commonly found throughout Europe. It typically grows near oak (*Quercus* sp.), chestnut (*Castanea sativa*), beech (*Fagus sylvatica*), and hornbeam (*Carpinus betulus*), forming symbiotic relationships with these trees. This species features a characteristic light brown stem and a brown cap. Valued by mushroom pickers not only for its appearance but also for its taste and texture, *Boletus reticulatus* Schaeff is known for its high protein content, low fat content, and significant levels of polyunsaturated fatty acids and tocopherols [28,29]. The mushroom also exhibits antioxidant activity. The fruiting bodies of *Boletus reticulatus* Schaeff contain elements such as Cr, Cu, Fe, Hg, Ni, Pb, and Zn. However, the levels of heavy metals (Cd, Hg, and Pb) are below toxic levels, indicating a negligible risk of adverse effects on human health [28].

##### Polysaccharides

The 15 kDa water-soluble heteropolysaccharide (BRS-X) isolated from the fungus features a backbone of 1,6-chain α-D-galactose and 1,2,6-chain α-D-galactose, with a glucose to galactose ratio of 1:5. Cells cultured with BRS-X, including T lymphocytes, B lymphocytes, and macrophages (RAW264.7 cells), exhibited increased proliferation compared to controls (30.97%, 38.88%, and 104.32%, respectively, at a concentration of 20 μg/mL). Additionally, BRS-X stimulated B lymphocytes to secrete higher amounts of IgG, IgE, IgD, and IgM. In assays using the S180 sarcoma tumor line, BRS-X showed a 39.74% inhibition rate at a concentration of 10 μg/mL. In a mouse xenograft model of S180 tumors, a decrease in tumor growth was observed compared to the control, with an inhibition rate of 37.86%. The researchers demonstrated that BRS-X’s antitumor effect involve the MAPK and PI3K/Akt signaling pathways, with a significant reduction in the expression of VEGF and VEGFR proteins during treatment [29].

#### 2.1.3. *Leccinum scabrum*

**Figure 3:**
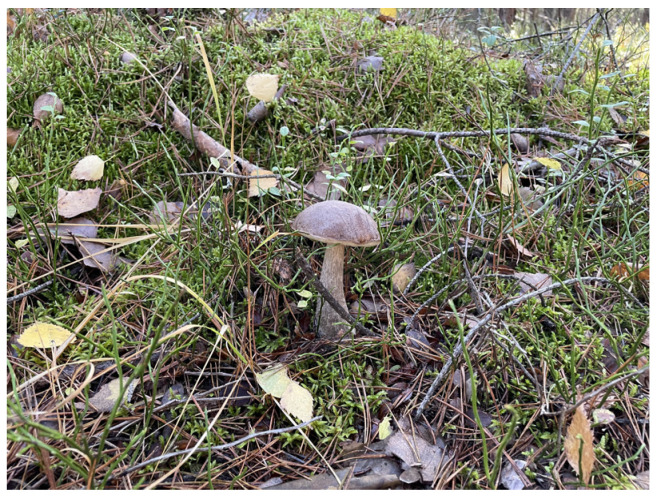
*Leccinum scabrum*.

*Leccinum scabrum* (Figure 3), also known as “Birch Boletus”, is a fungus commonly found in Central and Eastern Europe, Scandinavia, Australia, and New Zealand. It is typically located near birch trees, with which it forms mycorrhizae. This mushroom is characterized by its fibrous stem and soft cap, and its young specimens are the tastiest. This species is a good source of minerals, vitamins, flavonoids, and phenolic acids, giving it antioxidant properties [30,31].

##### Extracts

The acetone extract of the fungus *Leccinum scabrum* exhibits strong antioxidant and antimicrobial activities against various bacteria and fungi. In cell culture studies in vitro, antitumor activity was observed against the cervical cancer cell line HeLa, the colon cancer cell line LS174, and the lung cancer cell line A549. In contrast, no cytotoxic activity was demonstrated against the MRC-5 human lung fibroblast cell line (IC_50_: 73.64 μg/mL, 130.63 μg/mL, 76.82 μg/mL, and above 200 μg/mL, respectively). The extract of the *L. scabrum* fungus showed no genotoxic activity and exhibited protective properties against the effects of H_2_O_2_ [31].

#### 2.1.4. *Suillus granulatus*

This fungus species, also known as the “Weeping Bolete”, is found all over the world. It has white flesh that rapidly turns yellow when cut. This fungus is characterized by a mild taste and smell, making it a good candidate to be combined with other species to enhance flavor [32].

##### Extracts

Stojanova et al. analyzed two extracts—an aqueous extract and an ethanol extract—obtained from *Suillus granulatus*. More α- and β-glucans were observed in the aqueous extract than in the ethanol extract. Both extracts demonstrated moderate antitumor activity in the MTT assay. Against the cervical cancer cell line HeLa, the aqueous extract of *S. granulatus* exhibited a stronger effect, with an IC_50_ value of 0.60 mg/mL after 72 h compared to 0.71 mg/mL for the ethanol extract. Conversely, against the human hepatocellular carcinoma cell line HepG2, the ethanol extract was more active, with an IC_50_ value of 0.56 mg/mL, whereas the aqueous extract had an IC_50_ value of 0.75 mg/mL [32].

##### Small Molecules

**Figure 4:**
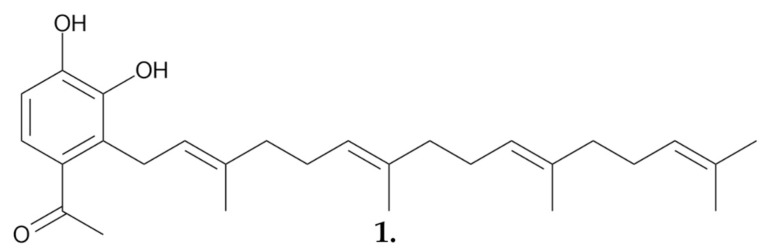
**Suillin (1)**, an anticancer compound obtained from dichloromethane extract *S. granulatus*.

Research on the obtained dichloromethane extract led to the isolation of a fraction containing the anticancer compound **suillin (1)** (Figure 4). This compound was active against human rhinopharyngeal cancer (KB), murine leukemia (*p*-388), and human bronchopulmonary carcinoma (NSCLC-N6) cell lines, with ID_50_ values of 0.69 μg/mL, 0.85 μg/mL, and 1.02 μg/mL, respectively. Additionally, the compound was tested in an in vivo model, yielding significant results using the *p*-388 cell line. The ratio of the average survival time of the test group to that of the control group was 151% at a dose of 5 mg/kg and 160% at 10 mg/kg [33].

**Figure 5:**
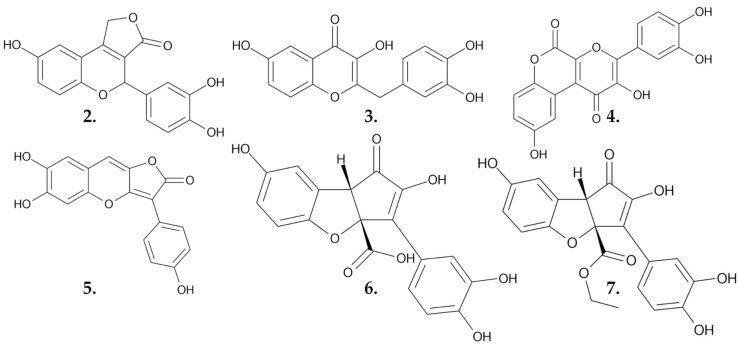
**Suillusol A–D (2–5)**, **suillusinoic acid (6)**, and **ethyl suillusinoate (7)** in sequence; polyphenolic metabolites extracted from *Suillus granulatus.*

Zhao et al. isolated and confirmed six novel polyphenolic metabolites from the fungus *Suillus granulatus* using high-resolution electrospray ionization mass spectrometry, nuclear magnetic resonance, and single crystal X-ray analysis. The anticancer potential of these compounds was assessed after 72 h of treatment of the HepG2 liver cancer cell line. The IC_50_ values of the newly discovered compounds ranged from 10 to 64 µM (Table 1). **Suillusol B (3)** (Figure 5) was identified as the most active compound, with an IC_50_ of 10.85 µM. The isolated compounds do not have a known role in the fungus’s biology; however, **Suillusol C (5)** (Figure 5) may represent a potential new type of fungal pigment [34].

#### 2.1.5. *Suillus luteus*

**Figure 6:**
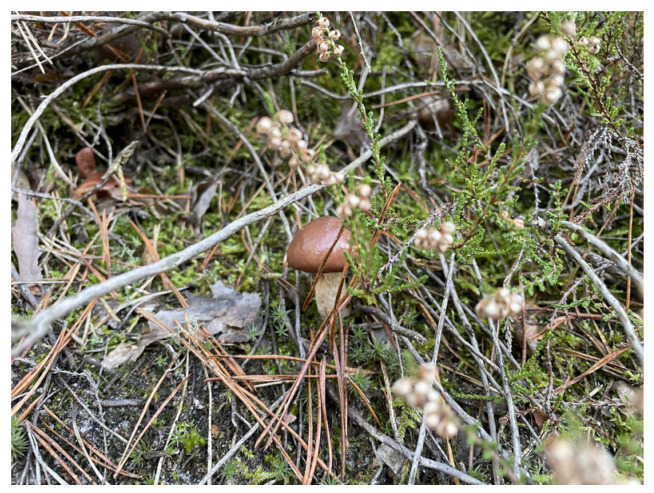
*Suillus luteus*.

*Suillus luteus*, commonly known as “Slippery Jack” (Figure 6), is frequently found in Central European forests, particularly near pines (*Pinus sylvestris*), spruces (*Picea abies*), and larches (*Larix decidua*), with which it forms symbiotic relationships. The mushroom is named “Slippery Jack” due to its distinctive mucilaginous skin on its cap, which should be removed before preparing the mushroom for consumption. *Suillus luteus* is a popular choice among mushroom pickers and is a good source of B vitamins and antioxidant compounds. Additionally, research has shown that the mercury content in this mushroom is so low that its consumption poses no risk to consumers [35,36].

##### Extracts

The ethanolic extract contains 0.606 µg/mL β-carotene and 0.357 µg/mL lycopene, while the methanolic extract contains 0.220 µg/mL β-carotene and 0.120 µg/mL lycopene.

Both the ethanolic and methanolic extracts of the mushroom *Suillus luteus* exhibit antimicrobial, antioxidant, and iron ion-chelating activities. In particular, the methanolic extract demonstrated notable antitumor activity in MTT assays on the breast cancer cell line MCF-7, with an IC_50_ value of approximately 173 µg/mL [37].

Another research group also analyzed phenol-rich extracts (ethanolic and methanolic), as well as polysaccharide extracts (from boiled water), for their anticancer activity and mechanisms. The methanol extract was found to be active after 48 h of exposure, with GI_50_ values of 30.33 µg/mL against lung cancer NCI-H460 cells, 30.30 µg/mL against gastric cancer AGS cells, 32.75 µg/mL against breast cancer MCF-7 cells, and 17.75 µg/mL against colon cancer HCT-15 cells. This extract showed the highest activity against cancer cells, primarily inhibiting the cell cycle in G1 phase in both HCT-15 and NCI-H460 cells. In contrast, the programmed cell death pathway was not activated, as indicated by the TUNEL assay. An increase in p53 protein levels was observed, but no changes in the levels of its transactivated target proteins were detected. Elevated levels of the cellular protein *p*-H2A.X suggest DNA damage [38,39].

##### Small Molecules

**Figure 7:**
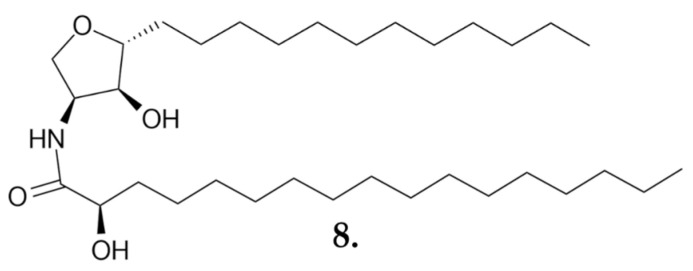
**Suillumide (8)**, a small-molecule phytosphingosine-type ceramide, is active against melanoma and breast cancer cell lines.

The structure of a phytosphingosine-type ceramide named **suillumide (8)** (Figure 7) was isolated from the ethanolic extract. The anticancer activities of suillumide and its acetyl derivative were then tested on the human melanoma cell line SK-MEL-1. In the MTT assay, after 72 h of treatment with the new compounds, IC_50_ values of 9.7 µM for suillumide and 11.6 µM for the acetyl derivative were obtained [40].

### 2.2. Order Agaricales

#### 2.2.1. *Armillaria mellea*

*Armillaria mellea*, commonly known as “Honey Mushroom”, is particularly popular in China, Eastern Europe, and the United Kingdom. In traditional Asian medicine, it is used alongside the orchid *Gastrodia elata*, with which it forms a symbiotic relationship. The medicinal product known as “Tianma” is used to treat headaches, dizziness, insomnia, and hypertension. Additionally, extracts from this mushroom are reported to possess a range of broad biological properties [41,42].

##### Polysaccharides

Wu et al. isolated and purified a water-soluble polysaccharide from the fruiting body of the fungus *Armillaria mellea* (AMP). The obtained AMP was characterized by homogeneity and had a molar mass of 4.6 × 10^5^ Da. Its composition included 94.8% sugars (mainly D-glucose), 2.3% uronic acid, and 0.5% proteins. AMP inhibited the growth of tumor cells in the human non-small-cell lung cancer line A549; at a concentration of 200 μg/mL, it inhibited about 50% of the cell population relative to the control. This effect was due to AMP’s ability to cause cell cycle arrest in G0/G1 phase and induce apoptosis through the loss of the mitochondrial membrane potential, release of cytochrome c, and activation of caspase-3 and -9 [43].

Nowacka-Jechalke et al. conducted a study of the crude polysaccharide fraction of the *Armillaria mellea* fungus (AmPS) obtained from natural habitats in Poland. They also concluded that this species is a rich source of polysaccharides (60.71% of AmPS), with a low presence of uronic acids, phenols, and proteins. The extract was mainly dominated by β-glucans. Further analysis revealed the presence of myo-inositol, mannitol, fucose, galactose, glucose, and mannose in the extract. AmPS exhibits potential antidiabetic, antioxidant, anti-inflammatory, and antitumor activities against gastric AGS, colon cancer HT-29, and lung cancer A549 cell lines. The activity was particularly high for gastrointestinal cancer cell lines, with IC_50_ values around 100 μg/mL after 72 h of AmPS incubation [44].

##### Small Molecules

**Figure 8:**
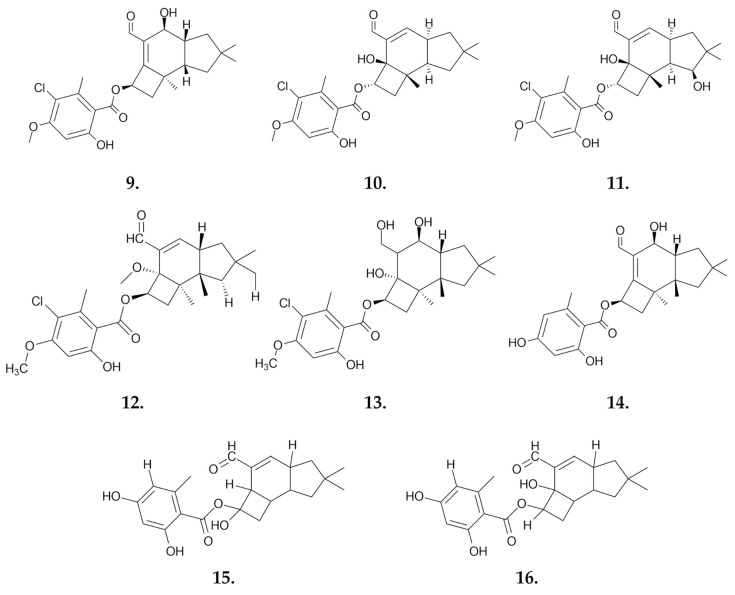
Sequentially, arnamial (**9**), armillaridin (**10**), armillarikin (**11**), 4-O-methylarmillaridin (**12**), 5′-methoxy-6′-chloroarmillane (**13**), dehydroarmillylorsellinate (**14**), 5-hydroxy-armillarivin (**15**) and melleolide (**16**)—small molecules obtained from *Armillaria mellea* with anticancer potential.

In 2009, Misiek et al. isolated a novel 2,4-protoilludane ester, **arnamial (9)** (Figure 8), from the *Armillaria mellea* fungus that demonstrated potent anticancer activity. This compound exhibited IC_50_ values of 10.69 μM against human intestinal cancer HCT-116 cells, 15.4 μM against breast adenocarcinoma MCF-7 cells, 3.93 μM against leukemia Jurkat cells, and 8.91 μM against the leukemia CCRF-CEM cell line in the MTT assay after 48 h of incubation. The cytotoxic activity of this compound was attributed to the activation of the programmed cell death (apoptosis) pathway, as evidenced by increased caspase-3 activity and DNA fragmentation [45].

**Armillaridin (AM) (10)** (Figure 8) is a sesquiterpenoid aromatic ester isolated from the mycelium of *Armillaria mellea* by Yang and colleagues in 1983 [46]. A colorless prism-shaped substance reduces the viability of leukemia cell lines K562 (chronic myeloid leukemia), U937, and HEL 92.1.7 (acute myeloid leukemia). After 48 h of incubation, the IC_50_ values were 4.4 μM, 3.0 μM, and 3.7 μM, respectively. Treated K562 cells exhibited characteristic changes indicative of autophagy, while U937 and HEL 92.1.7 cells showed strong features of apoptosis without autophagy. Armillaridin had no significant effect on the viability of CD14+ monocytes. The compound was confirmed to induce autophagy in K562 cells through autophagic flux, involving the induction of autophagosomes, fusion of autophagosomes with lysosomes, and downregulation of BNIP3 [41]. Armillaridin also exhibits potent cytotoxic activity against esophageal cancer cells CE81T/VGH, TE-2 (squamous cell carcinoma), BE-3, and SKGT-4 (adenocarcinoma), with IC_50_ values of 6.9 μM, 3.4 μM, 5.4 μM, and 5.5 μM, respectively. In these cell lines, armillaridin induces apoptosis and cell accumulation in G2/M phase of the cell cycle. An in vivo study involved administering armillaridin to animals with xenotransplanted CE81T/VGH tumors three times a week for a total of twelve doses at a concentration of 80 mg/kg body weight. Tumor growth arrest was observed with no decrease in body weight or white blood cell counts. AM also exhibits radiosensitizing properties in the CE81T/VGH cell line, with a sensitizer enhancement ratio of 1.6 [47]. Additionally, AM shows cytotoxic activity against the hepatocellular carcinoma (HCC) cell lines Huh7, HepG2 and HA22T/VGH (HA22T). The compound does not cause apoptotic death in liver cancer cell lines; instead, it induces cell death through autophagy, as evidenced by increased levels of ATG5, ATG7, beclin, and BNIP, and the induction of the LC3-I to LC3-II conversion [42].

Another substance with anticancer potential isolated from the fermented mycelium of *Armillaria mellea* is **armillarikin (11)** (Figure 8). This compound exhibits cytotoxic activity in vitro against K562 chronic myeloid leukemia cells and HL-60 and U937 acute myeloid leukemia cells, with IC_50_ values in the AlamarBlue^®^ assay of 12.2 μM, 10.8 μM, and 12.2 μM, respectively. The process of cell death proceeds through apoptosis, as confirmed by the accumulation of cells in sub-G1 phase of the cell cycle and decreases in the levels of procaspase-3, -8 and -9, as well as PARP. Additionally, the presence of the apoptotic phosphorylation marker H2A.X and increased levels of intracellular ROS were confirmed, supporting the mode of elimination of cancer cells [48]. Armillarikin also shows the ability to inhibit the growth of Huh7, HepG2 and HA22T hepatocellular carcinoma (HCC) cells. The compound exerts similar effects on HCC cells by inducing the apoptosis pathway, analogous to its effect on leukemia cell lines [49].

Bohnert et al. isolated five new derivatives of melleolides—sesquiterpene aryl esters **(12–14)** (Figure 8)—that are secondary products of fungi of the *Armillaria* genus. A group of these compounds were previously known for their antibiotic activities. The newly identified compounds (four out of five) showed cytotoxic activity against breast adenocarcinoma (MCF-7), leukemia (K-562, Jurkat) and cervical cancer (HeLa) cell lines (Table 2.). The authors of the study indicated that melleolides exert cytotoxicity mainly by inhibiting DNA biosynthesis [50].

Two novel protoilludane sesquiterpene derivatives **(15–16)** (Figure 8) were isolated from the mycelium of the fungus *Armillaria mellea*, **5′-methoxy-armillasin (15)** and **5-hydroxy-armillarivin (16)**, obtained from a chloroform extract. These colorless powders were identified alongside eight compounds with known structures. Most of the compounds tested showed activity against HepG2 liver cancer cells, with weaker activity against normal liver cells. Melleolide exhibited the highest anticancer activity against HepG2 cells with an IC_50_ of 4.95 μg/mL, while showing weaker activity against normal L02 liver cells, with an IC_50_ of 16.05 μg/mL. In contrast, 5′-methoxy-armillasin showed no activity, and 5-hydroxy-armillarivin had IC_50_ values of 18.03 μg/mL against HepG2 cells and 22.70 μg/mL against normal L02 cells. Melleolide significantly reduced tumor cell viability by affecting the levels of various proteins associated with apoptosis and cell proliferation, such as caspase-3, -8, -9, Bax and Ki67. Additionally, melleolide induced cell cycle arrest of HepG2 cells in G2/M phase [51].

#### 2.2.2. *Macrolepiota procera*

**Figure 9:**
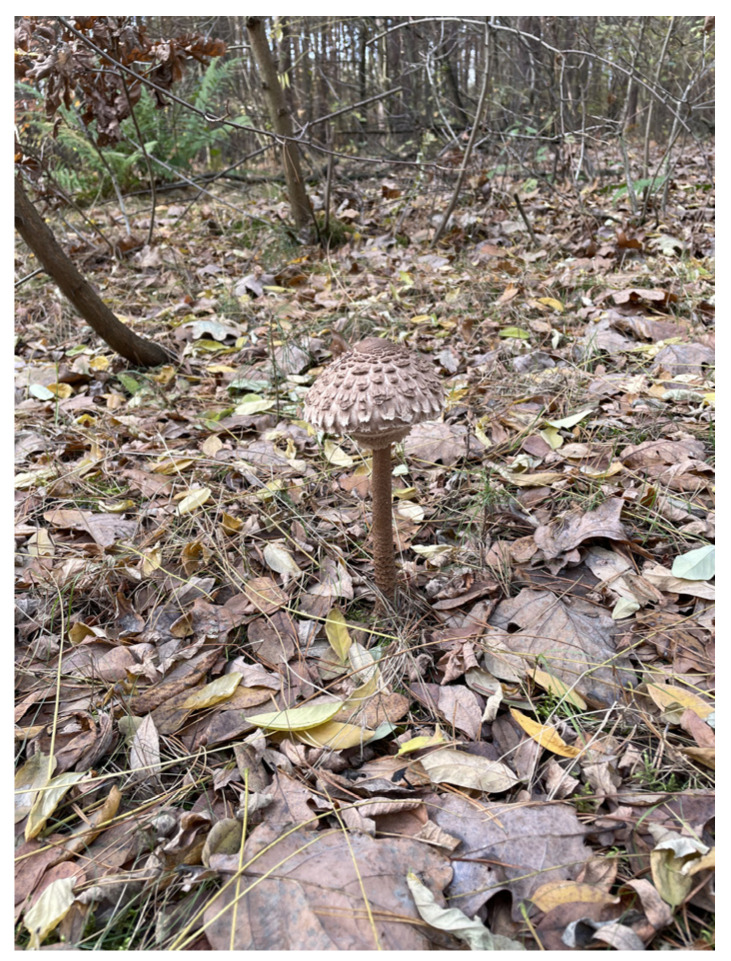
*Macrolepiota procera*.

*Macrolepiota procera* (Scop.) Singer (Figure 9), commonly known as the “Umbrella Mushroom”, belongs to the *Agaricaceae* family and is highly valued for its delicious flavor. The large fruiting body of *M. procera* resembles an umbrella, which explains its English name. Its cap features a snakeskin pattern and requires heat treatment before consumption, while the stem is inedible. Characterized by high levels of protein, carbohydrates, and dietary fiber, this mushroom has low levels of fat and energy [52,53]. Despite its culinary value, *M. procera* can accumulate varying amounts of minerals and toxic metals, depending on its environment. Studies have shown that mushrooms collected near roads can contain significant levels of toxic elements such as arsenic and cadmium, posing potential health risks. However, most studies have not found conclusive evidence questioning the safety of consuming these mushrooms, depending on their levels of contamination [53,54].

##### Extracts

The methanolic extract of the fruiting body of this species shows moderate antioxidant activity and antimicrobial activity. Additionally, the extract has demonstrated antitumor activity against HeLa epithelial carcinoma cells, A549 lung cancer cells, and LS174 colon cancer cells, with IC_50_ values of 29.39, 25.55, and 74.01 μg/mL, respectively. The mechanism of anticancer action for this fungus species has not yet been identified [27].

##### Small Molecules

The lanostane-type triterpenoids isolated from the umbrella mushroom exhibited significant inhibition of nitric oxide production in RAW 264.7 mouse macrophages in vitro, with the most potent compound showing an IC_50_ of 17.9 μM. Additionally, these triterpenoids demonstrated cytotoxic activity against human cancer cells, with IC_50_ values ranging from 3–25 μM across various cell lines: human myeloid leukemia HL-60, lung cancer A-549, human hepatocellular carcinoma SMMC-7721, breast cancer MCF-7, and human colon cancer SW480 cells. One of the compounds also showed weak antituberculosis activity (*Mycobacterium tuberculosis* H_37_Ra) [55].

#### 2.2.3. *Tricholoma equestre*

*Tricholoma equestre*, also known as *Tricholoma flavovirens*, is commonly known as the “Yellow Knight mushroom” or “Man on Horseback”. This fungus is widely distributed in Europe, North America, Central Asia, and Japan. *Tricholoma equestre* lives in symbiosis with coniferous trees, primarily pine and less commonly fir and spruce. The cap of *Tricholoma equestre* is broadly convex with a curled edge in young specimens, becoming almost flat in older ones. The color of the fruiting bodies changes from light yellow to yellow-green in the immature stage, turning olive-green with a brown or brownish-red tint as the mushroom ages. *Tricholoma equestre* is a controversial fungus, due to the lack of a clear scientific consensus on its safety for consumption. In small quantities, it is a source of essential nutrients, including bio-elements, fatty acids, and sterols. In some countries, it is considered poisonous, while in others, it is still widely harvested from the wild and consumed. There is a link between the consumption of large quantities of *Tricholoma equestre* fruiting bodies and the occurrence of rhabdomyolysis, a condition characterized by the rapid breakdown of striated muscle tissue. Symptoms of rhabdomyolysis include general weakness, muscle pain, sweating without fever, and dark-colored urine [56,57].

##### Extracts

The ethanol extract of this fungus species shows activity against two mouse cell lines, lymphoid leukemia L1210 and lung cancer 3LL cells, as well as human glioma U251 cells. The IC_50_ values for these lines are 56 μg/mL for L1210 cells, 18 μg/mL for 3LL cells, and 43.5 μg/mL for U251 cells [58].

##### Small Molecules

**Figure 10:**
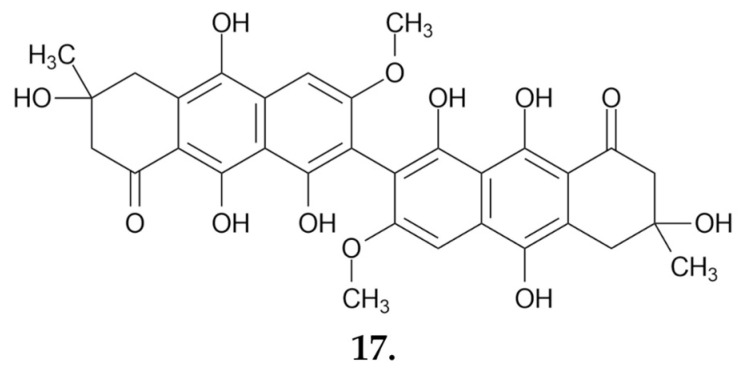
**Flavomannin-6, 6-dimethylether (17)**—a compound from *T. equestre* that affects human colon cancer cells by inducing apoptosis and inhibiting the cell cycle.

A bright yellow pigment, identified and confirmed by mass spectrometry and NMR spectroscopy as **flavomannin-6,6-dimethylether (17)** (Figure 10), was isolated as a pure compound. This compound inhibits the proliferation of human Caco-2 colon adenocarcinoma cells, with an IC_50_ value of 96 μg/mL after 24 h and 78 μg/mL after 48 h. The pigment also induces cell death, including apoptosis. Additionally, it inhibits the cell cycle in G0/G1 phase, as evidenced by an increase in the concentration of the p27 protein, which is associated with blocking cells in this phase [59].

#### 2.2.4. *Tricholoma terreum*

*Tricholoma terreum*, also known by various names such as “Grey Knight” or “Dirty Tricholoma” due to the discoloration on its blades, is an edible mushroom. It typically appears during the first cold weather. The mushroom has a light or dark gray cap, about 5 cm wide, covered with small gray scales. The stalk is relatively small and lacks a ring. The flesh of the mushroom is white-gray [60].

##### Extracts

Methanol and ethanol extracts of the fungus *Tricholoma terreum* were tested for their cytotoxic activity on the colon cancer cell line HT-29. The study showed that the methanol extract caused a 73% reduction in cell viability at a concentration of 250 µg/mL. The ethanol extract, on the other hand, had no significant effect on the viability of the HT-29 cell line [61].

##### Small Molecules

**Figure 11:**
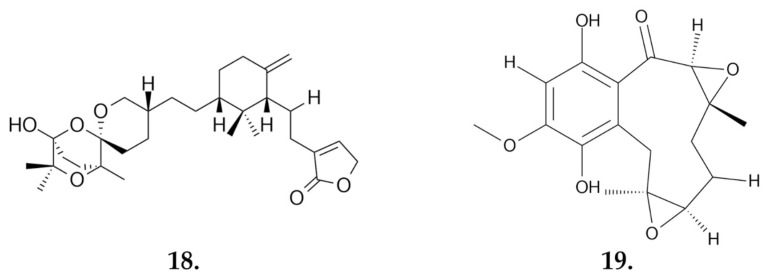
Anticancer active compounds derived from the fungus *Tricholoma terreum*: **saponaceolide Q (18)** and **terreumol A (19)**.

From the chloroform extract, according to the study by Feng et al., it was possible to isolate and confirm the structures of three triterpenoids: saponaceolides Q, R, and S. Studies on the cytotoxicity of these compounds were carried out using the MTT assay and various cell lines: human myeloid leukemia HL-60, hepatocellular carcinoma SMMC-7721, lung cancer A-549, breast cancer MCF-7, and colon cancer SW480 cells. Among the compounds tested, only **saponaceolide Q (18) (Figure 11**) showed cytotoxic activity. Its IC_50_ values, the concentration that inhibits cell growth by 50%, were as follows: 12.2 μM for HL-60 cells, 19.3 μM for SMMC-7721 cells, 12.2 μM for MCF-7 cells, and 1.4 μM for SW480 cells. For the A-549 cell line, the IC_50_ value was above 40 μM [62].

Meroterpenoids are compounds of biosynthetic origin containing a phenolic, quinone, or similar unit linked to a terpenoid by at least one C-C bond. A group of these compounds is distinguished by their strong biological activities. Four meroterpenoids, **terreumoles A-D (terreumol A, 19)** (Figure 11), with a 10-unit ring system, were isolated from the species *Tricholoma terreum*. Three of them were subjected to in vitro cytotoxicity tests for anticancer activity on the breast cancer MCF-7, hepatocellular carcinoma SMMC-7721, human myeloid leukemia HL-60, colon cancer SW480 and lung cancer A-549 cell lines (Table 3) [63]. Additionally, synthetic modifications of the 10-unit ring of the natural product terreumol C through oxygenation and bromination led to a series of new analogs of the compound. Several of these new compounds showed cytotoxicity at levels similar to the starting product in studies on the KB-3-1 cervical cancer and MCF-7 breast cancer cell lines [64].

### 2.3. Order Cantharellales

#### 2.3.1. *Cantharellus cibarius*

**Figure 12:**
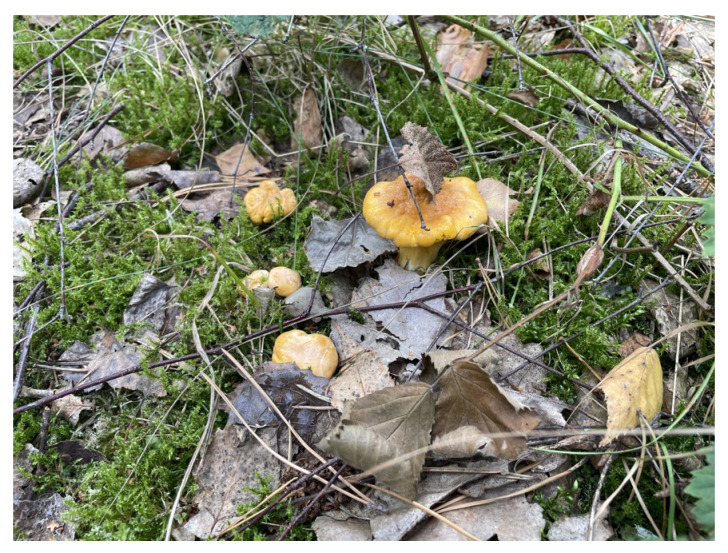
*Cantharellus cibarius*.

*Cantharellus cibarius* (Figure 12) also known as the “Golden Chanterelle”, is a widely distributed mushroom species found in Asia, Europe, and Central America. It thrives in symbiotic relationships with trees such as spruce, oak, and hornbeam. The fruiting bodies of *Cantharellus cibarius* are highly prized for their taste, aroma, and texture, making them one of the most popular mushrooms among Polish mushroom pickers [65].

##### Polysaccharides

Lemieszek et al. isolated a water-soluble fraction of small RNAs from the fruiting body of the fungus *C. cibarius*, which exhibited potent antiproliferative and proapoptotic activities. In the MTT assay, the small RNA fraction achieved IC_50_ values of 11.1 μg/mL and 2.6 μg/mL against the human colon adenocarcinoma HT-29 and LS180 cell lines, respectively, after 96 h of exposure. In contrast, the human colon epithelial CD841 CoTr cell line showed no sensitivity to the small RNAs. The antiproliferative activity was confirmed by the BrdU assay, where the IC_50_ for DNA synthesis inhibition in the LS180 cell line was 6.0 μg/mL. Additionally, cell cycle arrest of LS180 cells in G0/G1 phase was observed, as confirmed by the western blot analysis showing decreased levels of Cyclin D1, CDK4, and CDK6. Apoptotic activity was confirmed by Hoechst 33342 and propidium iodide (PI) double staining. The expression of negative cell cycle regulators, p53 and p21 proteins, was increased, and gene silencing of these proteins resulted in increased proliferation of LS180 cells [66].

Nowacka-Jechalke et al. isolated a polysaccharide fraction from the fruiting body of *C. cibarius* characterized by repeating units of →6)-α-D-Manp- (1→ monosaccharides. This fraction exhibited broad biological activity, including anti-inflammatory and prebiotic properties. In terms of anticancer activity, the polysaccharide fraction inhibited the proliferation of the LS180 cell line by 20% at a concentration of 100 μg/mL after 96 h of incubation. Notably, the fraction had no significant effect on the CCD841 CoTr cell line, indicating its selective activity against cancer cells [67].

Further studies on polysaccharide fractions isolated from *C. cibarius* revealed notable biological activities and mechanisms of action. Three fractions were examined: CC1, CC2b, and CC2a. Among these, the CC2a fraction, which is a polysaccharide-glycoprotein predominantly composed of branched mannan, demonstrated the most significant effects. This fraction showed substantial cytotoxicity against the LS180 cell line, with CC_50_ and IC_50_ values of 1957 μg/mL and 206 μg/mL in the LDH and BrdU assays, respectively, while it had a lesser impact on CCD841 CoN cells. The CC2a fraction induced cell cycle arrest in G0/G1 and S phases, resulting in increased DNA fragmentation and reduced tumor cell motility. The antiproliferative effects were linked to the modulation of the NF-κB pathway, influencing various target genes, including BAX, BCL2, CCND1, BIRC5, MYC, and MMP9 [68]. Additionally, the CC2a fraction enhanced the viability and proliferation of NK92 (human natural killer) cells by increasing the expression and translation of the AKT1, CREB1 (cAMP response element binding protein), MAPK3 (Erk1), and MAPK14 (p38) proteins. Adding NK cells to adherent cell lines LS180 and A549 enhanced the antitumor activity of the CC2a fraction. This effect was due to both the stimulation of NK cells and the direct antitumor action on the adherent cells. However, this intervention did not affect the normal intestinal cell line [69].

##### Extracts

A group of researchers obtained and tested four extracts of the fruiting body of *C. cibarius*: cyclohexane, dichloromethane, methanol, and water extracts. The extracts were tested for anticancer activity on the HeLa (human epithelial cervical cancer cells), NCI-N87 (human gastric carcinoma cells) and MRC-5 (human embryonic lung fibroblast cells) cell lines. Among the extracts, only the cyclohexane and dichloromethane extracts exhibited cytotoxic activity. The dichloromethane extract showed stronger activity against the HeLa cell line, while it was less active against the normal MRC-5 cell line compared to the cyclohexane extract. The IC_50_ values were 57.40 μg/mL for HeLa cells, 69.35 μg/mL for NCI-N87 cells, and 86.98 μg/mL for MRC-5 cells for cyclohexane extract. For the dichloromethane extract, the IC_50_ values were 39.26 μg/mL for HeLa cells, 63.46 μg/mL for NCI-N87 cells, and 134.79 μg/mL for MRC-5 cells [70].

### 2.4. Order Russulales

#### 2.4.1. *Lactarius deliciosus*

**Figure 13:**
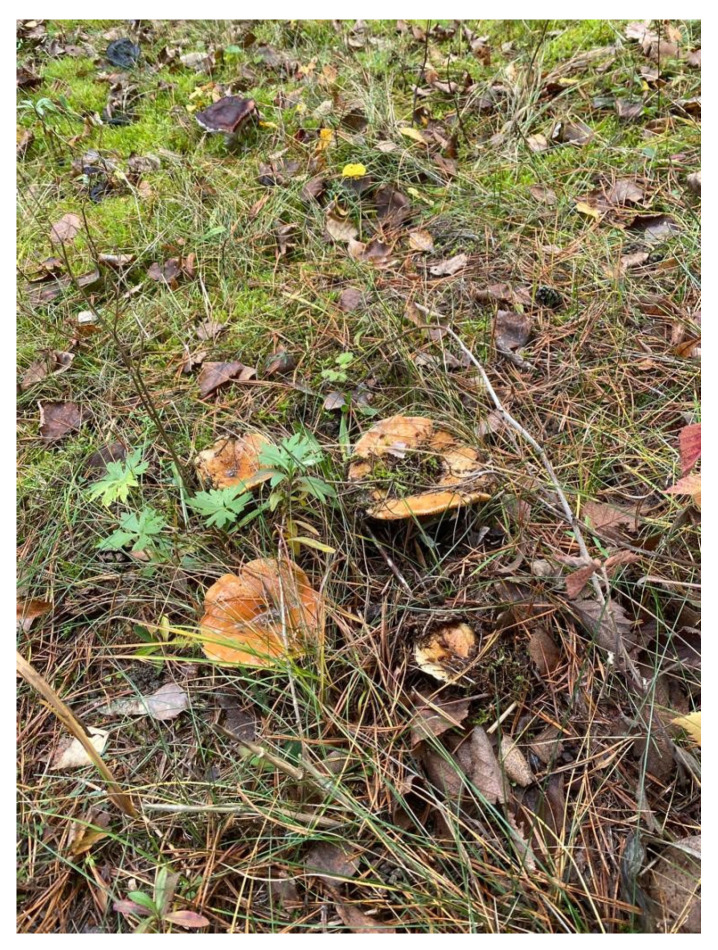
*Lactarius deliciosus*.

*Lactarius deliciosus* (Figure 13), commonly known as the “Saf Milk Cap”, is a highly popular edible ectomycorrhizal mushroom. Found in tropical, subtropical, and temperate forests, these mushrooms are easily recognizable by their vibrant orange latex, which is rich in pigments. They have distinct zoned caps and stems with cavities. Valued for their flavor and texture, *Lactarius deliciosus* mushrooms are not only appreciated for their culinary uses but also for their numerous health benefits. These include antioxidant, anti-inflammatory, antimicrobial, antihyperglycemic, immunomodulatory, and anticancer activities [71,72].

##### Oligosaccharides

An oligosaccharide from the fruiting body of the fungus *Lactarius deliciosus* named LDGO-A has a mass of approximately 945 Da and is composed of D-glucose and D-xylose. Similar to LDG-A, LDGO-A exhibits immune-stimulating activity and inhibitory effects against S180 tumors in an in vivo mouse sarcoma model. When administered at a dose of 40 mg/kg, LDGO-A achieves a 40.02% reduction in tumor size compared to the control. This oligosaccharide does not affect internal organs except for causing enlargement of the thymus and spleen, which is attributed to its immune-stimulating properties [73].

##### Polysaccharides

Ding et al. isolated a new polysaccharide named LDG-A from the fruiting bodies of *Lactarius deliciosus*. The molecular weight of LDG-A is estimated to be 11 kDa. Its structure is characterized by a predominance of L-mannose and D-xylose in a 3:1 ratio. The polysaccharide backbone consists of 1,6-linked L-mannopyranose units with branching at O-2, and the branches are mainly composed of D-xylopyranose residues linked by a →3)- bond. LDG-A demonstrated inhibitory activity against the murine sarcoma cancer S180 cell line in a mouse xenograft model, with a tumor inhibition rate of 68.42% at a dose of 80 mg/kg. Lower doses showed relatively weaker effects. Notably, at a dose of 40 mg/kg, the weights of spleen and thymus were significantly higher compared to the control group, suggesting that the polysaccharide might stimulate the immune response as part of its antitumor mechanism [74]. Further research supported this hypothesis, showing that LDG-A significantly promotes spleen cell proliferation, enhances the phagocytosis of mouse peritoneal macrophages, increases the expression of the TNF-α, IL-6, and iNOS genes, and increases the levels of the corresponding cytokines. These findings highlight the potential of *Lactarius deliciosus* as a source of natural immunostimulants [75].

##### Extracts

The methanol extract of *Lactarius deliciosus* is notably rich in essential minerals, including iron and zinc. It also contains copper, manganese, nickel, cadmium, lead, chromium, and cobalt. This extract demonstrates significant antioxidant properties and antimicrobial activity against various microorganisms. In vitro testing with the methanol extract revealed significant anticancer activity, inhibiting cervical cancer HeLa, non-small cell lung cancer A549, and colon cancer LS174 cells in the MTT assay with IC_50_ values of 19.01, 33.05, and 74.01 μg/mL, respectively [27].

Aqueous (RW), 70% ethanol (RE70), and 95% ethanol (RE95) extracts of the fruiting body of the fungus *Lactarius deliciosus* were evaluated for cytotoxic effects on normal human astroglial SVGp12 cells and human glioma U87MG and LN-18 cell lines after 48 h of exposure. The aqueous extract was inactive against the U87MG cell line within the tested concentration range. In contrast, the 70% ethanol extract (RE70) and 95% ethanol extract (RE95) both showed significant cytotoxicity. At a concentration of 250 µg/mL, RE70 reduced cell survival to about 50% of the control, while RE95 reduced it to approximately 20%. For the LN-18 cell line, the same concentration resulted in cell survival rates of about 55% (RW), 70% (RE70), and 35% (RE95). For normal SVGp12 astroglial cells, the survival rates were about 70% (RW), 50% (RE70) and 15% (RE95), respectively. All tested extracts caused a decrease in DNA biosynthesis in the tested cell lines, with the most pronounced effect observed for RE95, followed by RE70 and RW. Additionally, the 95% ethanol extract notably affected the activities of MMP-2 and MMP-9 enzymes, suggesting potential anticancer properties [72].

## 3. The Scientific Literature Lacks Reports on the Anticancer Activity of Many Edible Fungi Commonly Found in Polish Forests

Although many mushrooms are widely harvested and valued for their flavor, their medical potential remains untapped. It is possible that future research could uncover previously unknown bioactive properties of these species (Table 4); however, now, there are no scientific data to indicate their abilities to counteract cancer.

## 4. Limitations of the Use of Edible Mushrooms Compared to Anticancer Agents

Mushrooms have been employed in traditional medicine across a multitude of cultures for centuries and lauded for their potential health benefits and healing properties. Nevertheless, despite their popularity and the growing interest in their medicinal use, mushrooms are not always the optimal or most reliable source for medicinal treatments. Several factors contribute to this limitation.

One of the primary challenges associated with the utilization of mushrooms as a source of medicine is the considerable variation observed in their nutritional and medicinal compositions. The same species of mushroom may exhibit varying levels of bioactive compounds, depending on the geographical location of cultivation. This variability presents a significant challenge in standardizing mushrooms for medicinal use, as it is difficult to guarantee that each mushroom contains consistent levels of the active ingredients necessary for effective treatment. This lack of consistency can result in less effective treatments or, in some cases, completely ineffective treatments, depending on the source of the mushrooms used [76].

A further significant issue with the utilization of mushrooms for therapeutic purposes is the potential for contamination. It is established that mushrooms are capable of absorbing and accumulating a range of substances from their surrounding environment. These include beneficial and harmful elements, such as pollutants and heavy metals. This phenomenon, designated as bioaccumulation, signifies that mushrooms developing in regions characterized by elevated contamination levels may become contaminated with toxic substances. For example, mushrooms that are cultivated in close proximity to industrial zones, roadways, or sources of contaminated water may exhibit elevated concentrations of heavy metals, including lead, cadmium, and mercury, which could pose a significant health risk. The potential for contamination is particularly concerning in the context of medicinal use, where the goal is to promote healing and improve health. If mushrooms are contaminated, the expected health benefits may be negated or even the opposite effect may be observed, whereby the mushrooms may exacerbate existing health problems or introduce new ones. This uncertainty adds another layer of risk to using mushrooms as a medicinal resource [77].

Furthermore, the issue of toxicity represents a significant challenge for the medicinal use of mushrooms. The toxicity of mushrooms can vary significantly even within the same species, depending on environmental factors. For example, certain mushrooms that are harmless or even beneficial in one region may be toxic in another due to differences in soil composition, climate, or other environmental factors. This variability in toxicity makes it challenging to determine which mushrooms are safe to use for medicinal purposes. Consequently, while mushrooms may still have a role to play in complementary and alternative medicine, their use should be approached with caution [56].

## 5. Conclusions

**Figure 14:**
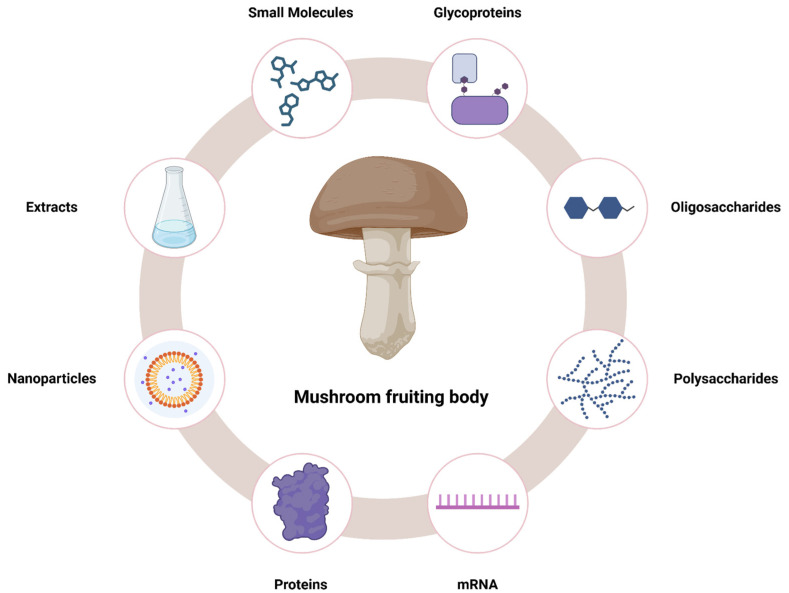
Summary of mushroom products with potential anticancer effects.

Mushroom fruiting bodies are a valuable source of various products, including extracts, polysaccharides, proteins, small molecules, and nanoparticles, all of which exhibit a wide range of biological properties, including anticancer potential (Figure 14). Scientific studies have demonstrated that these compounds can induce a variety of mechanisms of action, such as the activation of apoptosis, autophagy, cell cycle arrest, DNA damage, and increased immune system activity. Due to these mechanisms, products extracted from mushroom fruiting bodies hold great therapeutic potential and represent a promising direction in the search for new anticancer therapies, especially mushrooms from regions like Roztocze, which is well preserved from pollution and industry. Moreover, their natural origin and the variety of bioactive components suggest they can be less toxic and more specific in their effects compared to traditional chemotherapeutic agents, highlighting their value in modern medicine. Nevertheless, the route to the practical therapeutic utilization of individual mushroom constituents is lengthy, largely due to the issue of standardization of mushrooms and the potential for heavy metal contamination. Furthermore, the majority of extracts and compounds have been tested in vitro, which represents the initial stage of preclinical studies. This approach carries a high risk of failure in animals. It is reasonable to suggest that mushroom extracts could be introduced as supportive therapy at an earlier stage.

## Figures and Tables

**Table 1:** IC50 values of small molecules derived from *Suillus granulatus* against the HepG2 liver cancer cell line.

Compound	IC_50_ Values [µM], 72 h
**Suillusol A (1)**	35.60
**Suillusol B (2)**	10.85
**Suillusol C (3)**	32.62
**Suillusol D (4)**	63.68
**Suillusinoic acid (5)**	56.78
**Ethyl suillusinoate (6)**	61.89

**Table 2:** IC_50_ values [μM] of the compounds isolated and identified by Bohnert et al. [50].

Compound/Cell Line	4-O-Methylarmillaridin	5′-Methoxy-6′-Chloroarmillane	Dehydroarmillylorsellinate	6′-Chloro-13-Hydroxydihydro-Melleolide
MCF-7	6.7	26.5	8.0	>100
Jurkat	4.1	13.3	16.9	46.6
HeLa	18.8	35.8	15.2	>100
K-562	20.6	25.0	5.0	>100

**Table 3:** IC_50_ values [μM] from the MTT assay for meroterpenoids obtained from *Tricholoma terreum.*

IC_50_ Values [µM], 48 h Exposures					
Compound	MCF-7	SMMC-7221	A-549	HL-60	SW480
**Terreumol A (19)**	4.0	11.3	4.2	16.1	14.2
**Terreumol C**	5.6	24.7	16.4	24.6	>40
**Terreumol D**	17.8	14.9	6.5	21	21.1

**Table 4:** The orders and species of edible mushrooms from Poland that currently have no reports of anticancer activity.

	Order	Species
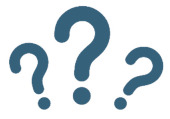	*Boletales*	*Leccinum aurantiacum*
	*Boletales*	*Leccinum griseum*
	*Boletales*	*Leccinum versipelle*
	*Boletales*	*Suillus variegatus*
	*Boletales*	*Suillus bovinus*
	*Boletales*	*Suillus grevillei*
	*Boletales*	*Imleria badia*
	*Boletales*	*Xerocomus subtomentosus*
	*Boletales*	*Xerocomellus chrysenteron*
	*Agaricales*	*Cortinarius caperatus*

## Data Availability

No new data were created or analyzed in this study. Data sharing is not applicable to this article.
